# Successful Surgical Treatment of Coronary Aneurysm, Ascending Aortic Aneurysm, and Bicuspid Aorta for a Kawasaki Disease Patient

**DOI:** 10.21470/1678-9741-2021-0056

**Published:** 2022

**Authors:** Ferhat Borulu, Yavuzer Koza, Bilgehan Erkut

**Affiliations:** 1 Department of Cardiovascular Surgery, Medical Faculty, Atatürk University, Erzurum, Turkey.; 2 Department of Cardiology, Medical Faculty, Atatürk University, Erzurum, Turkey.

**Keywords:** Mucocutaneous Lymph Node Syndrome, Coronary Aneurysm, Aortic Aneurysm, Bicuspid Aorta, Coronary Angiography, Morbidity

## Abstract

Kawasaki disease was first reported in 1967, and it was classified as an autoimmune vasculitis of the small and medium arteries. It is a self-limiting condition that occurs mostly in childhood, but it may involve complications — such as coronary artery aneurysms, myocardial ischemia, and arrhythmias — with significant morbidity and mortality that occur later in life. In this article, we present the association of an ascending aortic aneurysm with bicuspid aortic disease in addition to coronary aneurysm in a 55-year-old patient diagnosed with Kawasaki disease.

**Table:** 

Abbreviations, acronyms & symbols	
LAD	= Left anterior descending coronary artery
RCA	= Right coronary artery

## INTRODUCTION

Kawasaki disease, which can cause coronary artery aneurysm, is a disease of uncertain etiology that can affect patients from an early age^[1,2]^. The condition progresses with significant morbidities toward the adult stages of life, and it can develop long-term sequelae^[1]^. In this article, we discuss treating this disease, emphasizing the treatment of developing coronary artery aneurysm, and successful surgery of the emerging ascending aortic aneurysm and bicuspid aortic valve.

Currently, no consensus has been established on the treatment of coronary artery aneurysms or coronary artery aneurysms with multiple systemic aneurysms. Treatment strategies depend on the presence and extent of coronary artery stenosis, the etiology of coronary artery aneurysms, patients’ age, and accompanying comorbidities^[1,2]^. The main preferred treatments are surgical coronary artery bypass grafting and aneurysm resection or ligation, percutaneous interventions, or conservative drug therapy (anticoagulants and etiological therapy)^[3]^. In our literature review, we found no Kawasaki disease cases involving this type of aneurysmatic large-vessel disease and valvular pathology.

## CASE PRESENTATION

**Fig. 1:**
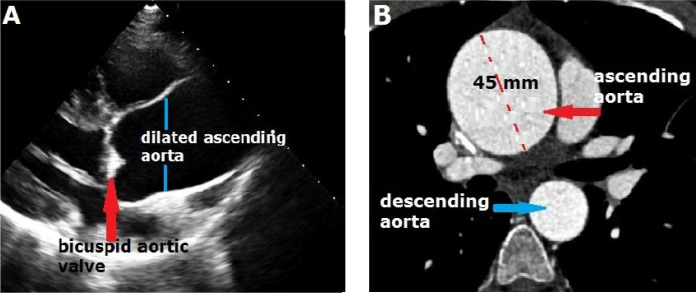
A) Ascending aortic aneurysm and bicuspid aortic valve in echocardiography (red arrow). B) Contrast-enhanced computed tomography shows the ascending aortic aneurysm approximately 45 mm wide (red arrow).

**Fig. 2:**
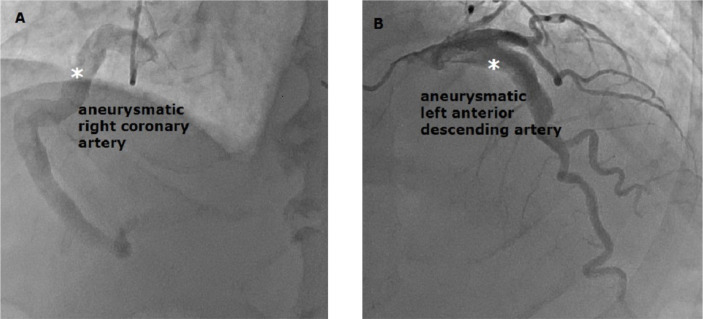
A, B) Right coronary artery and left anterior descending coronary artery angiography depicts coronary artery aneurysms (white asterisks).

**Fig. 3:**
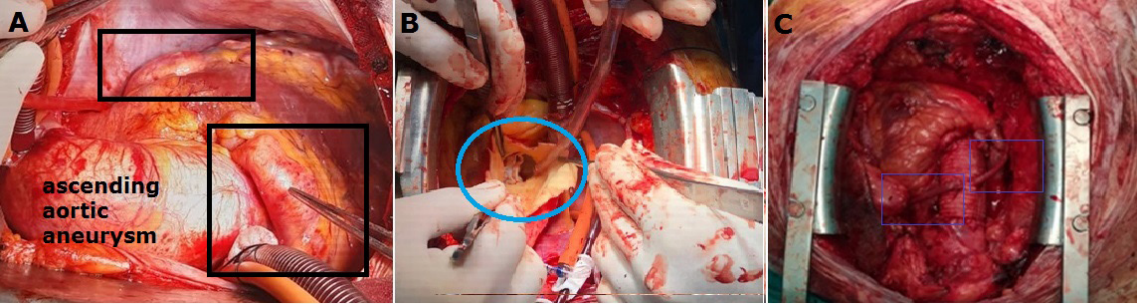
A) Ascending aortic aneurysm, right coronary artery (RCA) and left anterior descending coronary artery (LAD) aneurysms detected during surgical procedure (black frame). B) After aortotomy, surgical image showing the bicuspid aortic valve (blue frame). C) Surgical image showing saphenous vein bypasses made to the distal parts of the occluded RCA and LAD (blue frames).

A 55-year-old male patient from a rural area was admitted to our emergency department with complaints of chest pain, palpitations, and shortness of breath. In describing his family history, he explained that he had experienced a febrile illness during childhood and that he has Kawasaki disease. During his adulthood, he had frequently presented at the emergency department with chest pain, but his complaints have been determined to have made him uncomfortable for the first time. Additionally, he revealed that he had not received any treatment for this disease during and since his childhood. Transthoracic echocardiography showed a bicuspid calcified aortic valve with two cusps (right and left) with a dome opening and a dilated ascending aorta (Figure 1A). Also, moderate, eccentric aortic regurgitation was present. The patient’s aortic root diameter was 3.0 cm, but the ascending aortic diameter was 4.5 cm. The aortic valve area was 0.8 cm^2^, and the mean aortic valve gradient was 48 mmHg while the peak gradient was 75 mmHg. Computed tomography was performed to support a diagnosis of ascending aortic aneurysm; it showed an enlargement of the patient’s ascending aorta (approximately 45 mm; Figure1B). Surgery was considered because of the bicuspid aortic valve and ascending aortic aneurysm. Moreover, coronary angiography was performed to investigate the presence of coronary artery aneurysm due to the patient’s Kawasaki disease diagnosis or to confirm the presence of any coronary lesions. Aneurysmal dilatation was detected in the patient’s right coronary artery (RCA) and left anterior descending coronary artery (LAD) in coronary angiography (Figures 2A and 2B). The patient was operated upon for advanced aortic stenosis, as well as coronary artery and ascending aortic aneurysm. His pericardium was opened after sternotomy. In addition to the ascending aorta, aneurysmatic dilatation of the patient’s coronary arteries was seen (Figure 3A). A cardiopulmonary bypass was initiated with femoral arterial and right atrial venous cannulation. A cross-clamp was placed in the patient’s distal ascending aorta, and an aortotomy was performed. The bicuspid aortic valve was seen (Figure 3B). Since no enlargement had occurred in the sinus of Valsalva, we decided to separately perform aortic valve and ascending aortic replacements. Since the patient’s weight was 85 kg and his height was 175 cm, we found his body surface area to be 2.03 m^2^. An aortic valve size with a sufficient effective orifice area was determined to prevent a “patient-prosthesis mismatch”, according to the patient’s body surface area. Furthermore, after his bicuspid aortic valve was excised, his aortic orifice diameter was measured with a cylindrical scale. It was determined that the aortic valve number 23 was suitable for the patient. An aortic valve replacement was performed with a bileaflet no. 23 prosthetic mechanical valve (Sorin Biomedica ART23LNF, Italy). Then, the patient’s right and left coronary sinuses were checked. We observed that the coronary sinuses mouths were not wide; therefore, no procedure was performed on the patient’s coronary artery sinuses. The patient’s coronary aneurysms were treated by tying the RCA and LAD in the epicardial region from the proximal and distal parts. We performed saphenous vein bypass grafting in the distant regions of the patient’s ligated RCA and LAD (Figure 3C). We could not use the patient’s left internal mammary artery due to insufficient flow. The surgical procedure terminated with an ascending aorta replacement using 30-mm tubular woven Dacron prosthesis (UB Shield GraftTM, Ube Medical Co. Ltd., Tokyo, Japan). The proximal parts of the patient’s saphenous veins were sutured to a synthetic ascending aortic graft. The patient was weaned from cardiopulmonary bypass without any problems. He underwent an uneventful recovery in the intensive care unit.

## DISCUSSION

Kawasaki disease, a vasculitis that typically occurs during childhood, can often be self-limited, but it may cause coronary artery aneurysms (20% probability) due to the lack of an early diagnosis, untreated disease progress, and inadequate treatment. Simple physical examination findings during patients’ childhood (bilateral bulbar conjunctival injection, mouth-mucosa changes, strawberry tongue, redness of the palms or soles, edema and periungual desquamation in the hands or feet, polymorph rash, and cervical lymphadenopathy) can be diagnosed and treated^[4]^. Our patient had been diagnosed in childhood due to such possible clinical findings, but his disease has progressed because no treatment had been applied. Reports have suggested that the incidence of coronary artery aneurysm could be reduced through treatment with 80-100 mg/kg of aspirin, especially in addition to intravenous immunoglobulin^[4,5]^.

Coronary artery aneurysm is defined as a localized dilation of the coronary artery that exceeds 1.5-2.0 times the diameter of adjacent segments, and it occurs in 1-4% of coronary angiographies. The most common cause of coronary artery aneurysms is atherosclerosis^[6,7]^. In addition, malformations such as Kawasaki disease, an abnormal origin of the coronary arteries, and congenital vascular connective tissue diseases are among the other important etiologies^[7,8]^. Because Kawasaki disease is rare, and a limited number of studies have researched the condition, its pathogenesis, treatment, and prognosis have not been determined exactly^[7]^. A previous Kawasaki disease diagnosis was the etiological cause of our patient’s coronary artery aneurysm.

Patients with coronary artery aneurysms can be admitted to the hospital with different clinical complaints, but most patients are asymptomatic. Angina pectoris, myocardial infarction, sudden death, fistula formation, hemopericardium, tamponade, compression of the surrounding structures, congestive heart failure, and even a simple murmur may be among these complaints or clinical signs. In our patient’s case, general complaints could have indicated any kind of nonspecific cardiac pathology, and the coronary aneurysm diagnosis could be made after angiography. Coronary angiography is the gold standard exam in coronary artery aneurysm diagnosis. For our patient, we performed a diagnostic angiographic study primarily to investigate the presence of coronary aneurysm and occlusion. The patient was scheduled for additional cardiac surgery because of his Kawasaki disease diagnosis.

Currently, no consensus has been established on the clinical features, diagnostic method, and treatment of such cases. Medical treatment, surgical excision, coronary bypass grafting, and percutaneous interventions are among the treatment methods available for patients with coronary aneurysm. If there is another surgical indication for these patients in addition to coronary artery aneurysm (coronary atherosclerosis, valve stenosis, and aortic pathology), surgical intervention can also be performed for coronary artery aneurysm. However, without additional surgical indications, treating these patients poses a clinical dilemma for clinicians due to a lack of randomized trials and community recommendations. Patient management differs for patients who have frequently been admitted to an emergency department with coronary complaints and who had previously been diagnosed with Kawasaki disease. Without aneurysmal development, these patients should be followed up for atherosclerosis, and a stent can be placed if necessary. But for patients with coronary aneurysm, experience with stents is limited. Bypass grafting has been established as a superior treatment to percutaneous coronary intervention^[9]^. Patients who had undergone percutaneous coronary intervention have also been reported to require more frequent revascularization than patients who had undergone coronary artery bypass grafting^[10]^.

## CONCLUSION

In the medical literature, we had never previously encountered the pathological association that we have discussed in this case report. Moreover, clinicians can overlook Kawasaki disease diagnosis during childhood, and significant cardiovascular damage may develop over time. This clinical process and the disease’s progression may remain hidden until a patient reaches adulthood, or they may occur with some non-specific and negligible findings over the years. Guidelines and obligations to promote treatment after early diagnosis are important to reduce morbidity and mortality among these patients.
